# A Tissue Factor Bi-Specific T-Cell Engager Provides Effective Targeting and Cytotoxicity Against Cervical Cancer Cell Lines

**DOI:** 10.3390/ijms26188941

**Published:** 2025-09-13

**Authors:** Kyung-jun Lee, Gilhyang Kim, Booseong Seo, Soo Young Jeong, Hyeong Su Kim, Hye-Yon Cho, Sung Taek Park

**Affiliations:** 1 Institute of New Frontier Research Team, Hallym University, Chuncheon 24252, Republic of Korea; 2 Department of Pathology, Kangnam Sacred-Heart Hospital, Hallym University Medical Center, Hallym University College of Medicine, Seoul 07441, Republic of Korea; 3 Department of Pathology, Kangbuk Samsung Hospital, Sungkyunkwan University School of Medicine, Seoul 03181, Republic of Korea; 4 Department of Biology, Johns Hopkins University, 3400 N. Charles St., Baltimore, MD 21218, USA; 5 Department of Obstetrics and Gynecology, Kangnam Sacred-Heart Hospital, Hallym University Medical Center, Hallym University College of Medicine, Seoul 07441, Republic of Korea; 6 Division of Hemato-Oncology, Department of Internal Medicine, Kangnam Sacred-Heart Hospital, Hallym University Medical Center, Hallym University College of Medicine, Seoul 07441, Republic of Korea; 7 Department of Obstetrics and Gynecology, Dongtan Sacred-Heart Hospital, Hallym University Medical Center, Hallym University College of Medicine, Hwasungsi 18450, Republic of Korea

**Keywords:** bispecific T-cell engager, immunotherapy, tissue factor, cervical cancer, targeted therapy

## Abstract

Tissue factor (TF), also known as CD142, is a 47 kDa transmembrane glycoprotein belonging to the class II cytokine receptors superfamily. High expression of TF has been reported to be correlated with poor prognosis in various cancers. In this study, we aimed to clarify the cytotoxicity of bi-specific T-cell engagers (BiTE) targeting TF on cervical cancer cell lines. We designed and characterized the novel humanized BiTE targeting TF using an anti-human CD3 single-chain variable fragment (scFv) linked to human TF scFv. TF-Bite replication and potency were assessed in cervical cancer cell lines. The expression of the TF-BiTE and the activation and proliferation of T cells induced along with the T-cell-mediated cytotoxicity were evaluated by flow cytometry in vitro. TF expression was confirmed in SiHa, ME-180, and HeLa cell lines. The TF-BiTE showed potent TF-specific cytotoxicity and induced T-cell activation, proliferation, degranulation, and cytokine release. These effects were not observed in TF-negative control cells. Our findings support TF-BiTE as a promising therapeutic candidate for cervical cancer immunotherapy.

## 1. Introduction

Cervical cancer remains a significant global health problem, representing the fourth most common cancer in women worldwide. In 2025, an estimated 13,360 new cases and 4320 deaths were reported in the United States for 2025 [1]. Although human papillomavirus (HPV) vaccination offers preventative measures, cervical cancer still is a leading cause of cancer-related mortality in developing countries [1]. While early-stage cervical cancer has good prognosis with more than 90% of 5-year survival rate, this drastically diminishes to below 20% in advanced stages, highlighting the critical need for improved therapeutic strategies. Current standard treatment for locally advanced disease is concurrent chemoradiotherapy (CCRT) or combination chemotherapy with pembrolizumab, but which could only be indicated for PDO1-positive metastatic cervical cancer, underscoring the urgency for innovative treatment approaches [2].

T-cell-based immunotherapies, including chimeric antigen receptor (CAR) T-cell therapies and bispecific T-cell engagers (BiTEs), represent a promising frontier in cancer treatment. CAR T-cell therapy has demonstrated remarkable success against hematological malignancies, particularly in refractory B-cell acute lymphoblastic leukemia [3]. BiTEs offer a less complex and potentially more broadly applicable alternative, employing dual targeting to effectively recruit and activate T cells for tumor cell elimination, even in the context of low neoantigen burdens [4,5].

While blinatumomab, a CD19-targeting BiTE, has received FDA approval for B-cell acute lymphoblastic leukemia, and numerous BiTEs are currently in clinical trials for hematological malignancies [4,6], their efficacy in solid tumors has remained limited, as demonstrated by the less-than-optimal results observed with cibisatamab and tebentafusp [7,8]. This underscores the crucial need for novel BiTEs targeting alternative antigens or employing innovative formats to overcome these challenges.

Tissue factor (TF), a 47 kDa transmembrane glycoprotein also known as CD142, emerges as a compelling target. Its high expression in various cancers, coupled with its established correlation with poor prognosis [9], suggests its potential as a therapeutic target. While TF is typically confined to vascular and epithelial cells, its activation of factors VII (FVII) into FVIIa significantly contributes to tumor growth, angiogenesis, and metastasis [10].

Preclinical and clinical data have shown that blocking the TF:FVIIa pathway can effectively inhibit tumor growth, as evidenced by the significant cytotoxic effects and promising clinical trial results observed with tisotumab vedotin, a TF-targeting antibody-drug conjugate [11,12].

This study focuses on the development and characterization of a novel TF-targeting BiTE for the treatment of cervical cancer. In vitro studies were conducted to evaluate its ability to induce cytokine production and exert cytotoxic effects against cervical cancer cell lines.

## 2. Results

### 2.1. TF Expression in Cervical Cancer and Normal Human Tissues from Cervix

**Figure 1:**
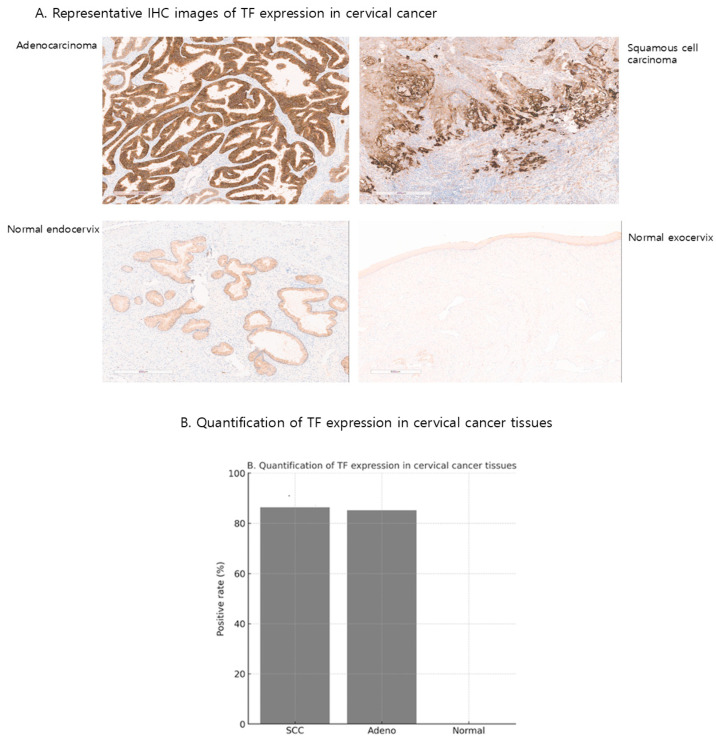
TF expression in cervical cancer and normal tissues. (**A**) Representative immunohistochemistry (IHC) images of TF expression in cervical squamous cell carcinoma, adenocarcinoma, and normal cervix. TF staining was detected in tumor tissues but absent in normal cervix. (**B**) Quantification of TF expressions in tissue samples. TF positivity was observed in 86.3% (145/163) of squamous cell carcinomas and 85.2% (58/68) of adenocarcinomas, whereas normal cervical tissues showed no detectable expression.

We transformed our TF scFv into a complete antibody, allowing us to evaluate TF expression in human tissue microarrays and resected tumors through immunohistochemistry. The same binding domain used in the TF BiTE was employed for this analysis. Our findings showed positive staining in cervical cancer specimens, indicating elevated TF expression, which was localized in the nucleus and cytoplasm. TF expression was observed in 86.3% (145/163) of cervical squamous cell carcinoma and 85.2% (58/68) of adenocarcinoma but was absent in normal cervical tissues. Representative IHC images are provided in Figure 1A. When comparing TF expressions between normal cervix tissues, including the endocervix and ectocervix, we observed that TF levels were higher in adenocarcinoma than in squamous cell carcinoma. (Figure 1B). Following our analysis of gene expression differences between tumor and normal cervix tissues, we concluded that targeting TF could represent an effective treatment strategy.

### 2.2. Design and Production of TF BiTE

#### 2.2.1. Schematic Diagram of TF-BiTE Structure

**Figure 2:**
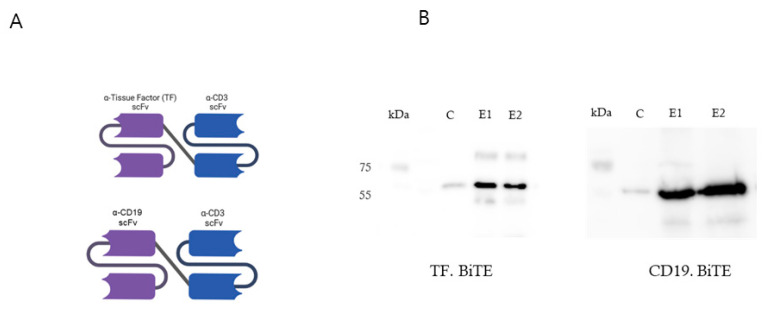
Structure and expression of TF-BiTE and control BiTE. (**A**) Schematic representation of the bispecific T-cell engagers (BiTEs). The TF-BiTE was constructed by genetically linking an scFv specific for tissue factor (TF) to an anti-CD3 scFv (clone OKT3) via a flexible (G_4_S)_3_ linker. The control BiTE was generated by replacing the TF scFv with an anti-CD19 scFv (clone FMC63). (**B**) Western blot analysis confirming the expression and size of TF-BiTE and CD19-BiTE proteins. Conditioned media from transfected HEK293T cells were collected, concentrated, and probed with anti-His antibody. Specific bands corresponding to the expected molecular weight (~55–60 kDa) were detected for both TF-BiTE and CD19-BiTE, whereas no signal was observed in control (C) lanes.

We identified a cluster of anti-TF antibodies within a rat hybridoma library that binds to either the distal membrane immunoglobulin-like (Ig) domain or the more proximal frizzled (Fr) domain of TF (Figure 2A). We successfully maintained the specificity of these antibodies for TF after converting them into a single-chain variable fragment (scFv) format. Our next step involved combining these scFvs with a murine CD3 scFv using a short Gly4Ser linker to create a TF BiTE molecule (Figure 2A).

We successfully expressed the BiTEs in HEK-293T cells using lentiviral vectors. The supernatant obtained from these cells underwent purification via metal-affinity chromatography, revealing a peak at approximately 40 mM imidazole. The purified product was then analyzed through SDS/PAGE electrophoresis, which displayed a dominant band at 55 kDa, consistent with the expected molecular weight of the BiTEs, as confirmed by Western blot analysis (Figure 2B).

#### 2.2.2. TF-BiTE Directs T Cells to Kill TF-Expressing Cervical Cancer Cells In Vitro

**Figure 3:**
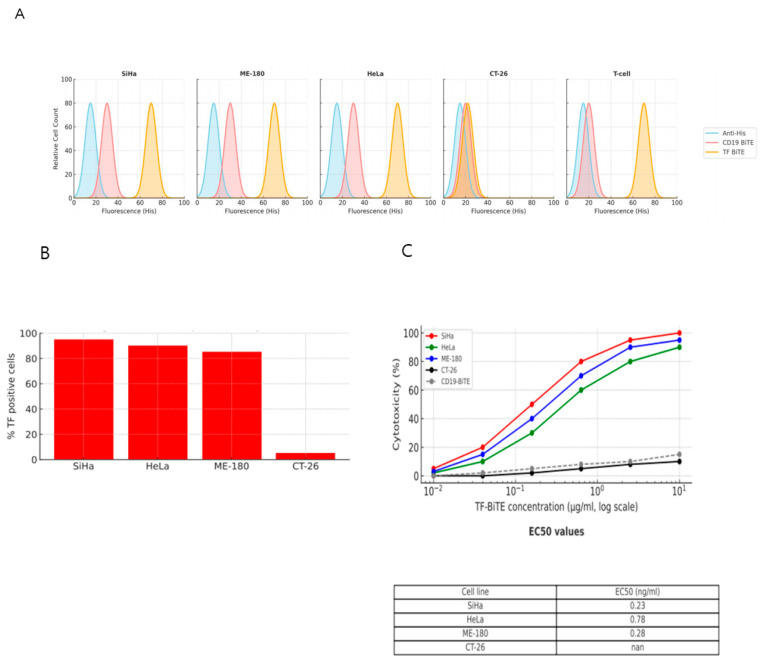
Correlation between TF Expression Level and Tumor Cell Lysis Activity of TF-BiTE. (**A**) Flow cytometry analysis showing specific binding of TF-BiTE to TF-positive cervical cancer cell lines (SiHa, HeLa, ME-180) and CD19-BiTE to human T cells. TF-negative CT26 cells showed no binding to either BiTE. (**B**) Cytotoxic activity of TF-BiTE against cervical cancer cell lines. Purified human T cells were co-cultured with TF-positive cervical cancer cell lines (SiHa, HeLa, ME-180) or TF-negative CT26 cells (effector-to-target ratio = 5:1) in the presence of TF-BiTE or CD19-BiTE for 36 h. TF-BiTE induced robust, antigen-specific tumor cell lysis in TF-expressing cervical cancer cells, while no effect was observed in CT26 cells. Data represent mean ± SEM from three independent experiments. (**C**) Quantification of tumor cell lysis under the same conditions as panel B. TF-BiTE treatment resulted in significant cytotoxicity against TF-expressing cervical cancer cells, whereas no lysis was detected in TF-negative CT26 cells. Data represent mean ± SEM from three independent experiments.

In this study, we assessed the ability of TF BiTE to induce antigen-specific cytotoxic responses against cervical cancer cell lines in vitro. The specificity of the TF BiTE was verified through flow cytometry staining tests performed on cervical cancer cell lines (SiHa, HeLa, and ME-180). Additionally, human T cells demonstrated specific binding to CD3 with both CD19 and TF BiTEs. In contrast, the murine colon carcinoma cell line (CT-26) showed no binding with either CD19 or TF BiTE (Figure 3A).

**Figure 4:**
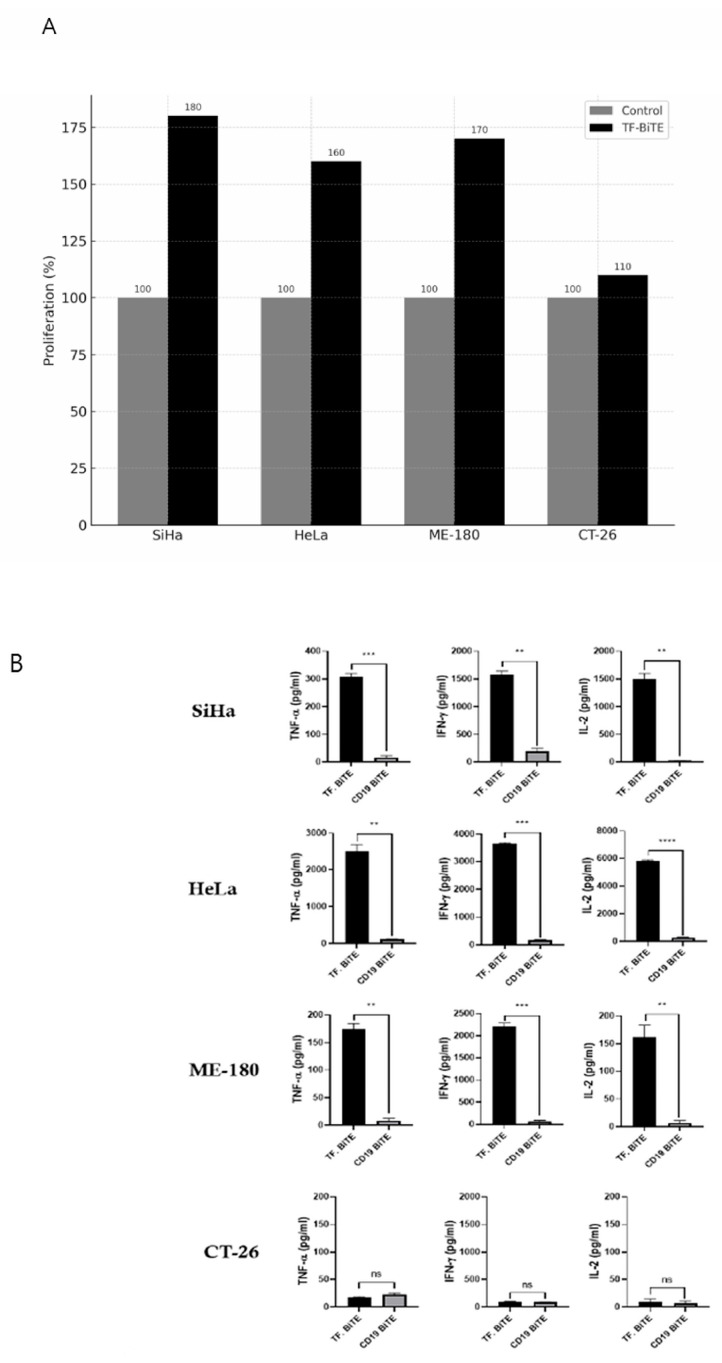
TF-BiTE–mediated T-cell activation and cytokine release. (**A**) Proliferation of T cells was evaluated after co-culture with TF-BiTE and different cervical cancer cell lines (SiHa, HeLa, ME-180) or the negative control cell line (CT-26). T-cell proliferation was measured by CFSE dilution and quantified as the percentage of proliferating T cells. TF-BiTE induced robust T-cell proliferation in the presence of TF-positive cervical cancer cells compared with control cultures. (**B**) ELISA measurement of TNF-α, IFN-γ, and IL-2 in supernatants collected from 24 h co-cultures of tumor cells and T cells (E:T ratio = 5:1) treated with TF-BiTE or control CD19-BiTE. TF-BiTE stimulation induced significantly higher cytokine secretion compared with CD19-BiTE, confirming functional T-cell activation. Error bars in all graphs and figures represent the mean value with the standard error of the mean from at least three independent experiments or assays. We used ns to indicate non-significant results, whereas ** *p* ≤ 0.01, *** *p* ≤ 0.001, and **** *p* ≤ 0.0001 indicate varying significance levels.

We co-cultured T cells with TF BiTE in conjunction with cervical cancer cell lines. Our findings revealed that only the cervical cancer cell lines exhibited cytotoxicity and cytokine secretion, confirming that the presence of TF is essential for T-cell activation (Figure 3B,C and Figure 4A,B). Additionally, there was no evidence of T-cell activation when TF BiTE was co-cultured with T cells in the TF-negative CT26 cell line (Figure 3B,C and Figure 4A,B).

### 2.3. Preliminary Mechanism Insights

#### 2.3.1. Proliferation Assay and ELISA

During the experiment, CD3^+^ T cells were co-cultured with various target cell lines and treated with either TF-BiTE or CD19-BiTE to evaluate the target specificity of TF-BiTE–mediated T-cell activation. A marked increase in CD3^+^/CFSE^−^ T cells was observed only in the presence of both TF-BiTE and cervical cancer cell lines, indicating robust T-cell proliferation (Figure 4A). The observed T-cell activation suggests that the TF-BiTE-induced interaction between T cells and TF-expressing tumor cells is essential for activation, rather than crosslinking of CD3 molecules alone.

Furthermore, cytokine release (TNF-α, IFN-γ, and IL-2), which is a hallmark of T-cell activation upon tumor cell lysis, was also induced by TF-BiTE in cervical cancer cell lines (Figure 4B). However, CT-26 did not show any T-cell activation or cytokine release by either TF-BiTE or CD19-BiTE. (Figure 4A,B).

#### 2.3.2. Activation Assay

**Figure 5:**
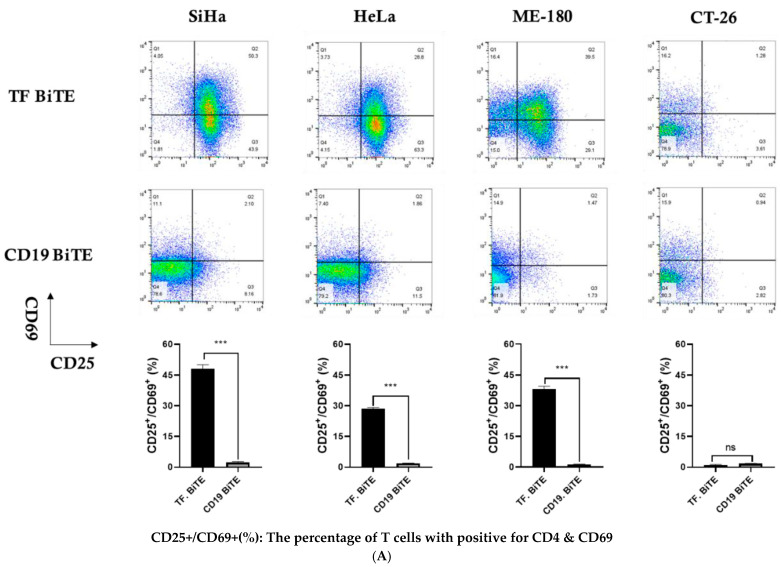

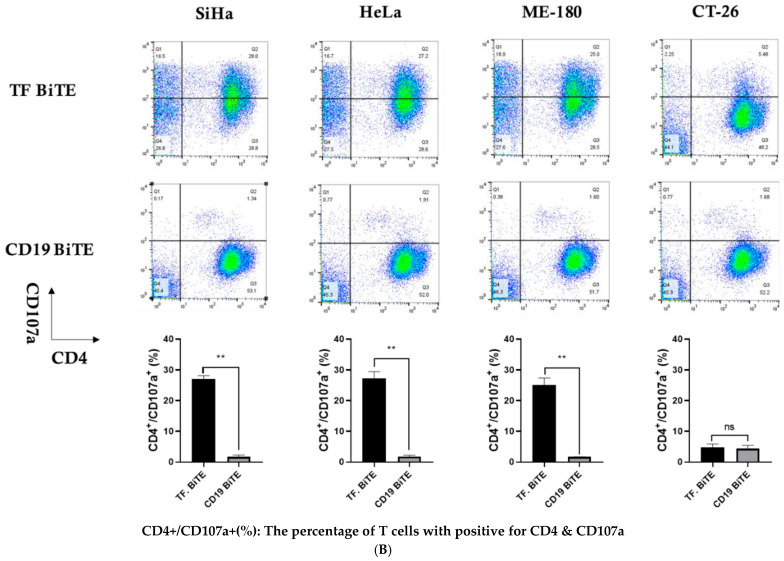

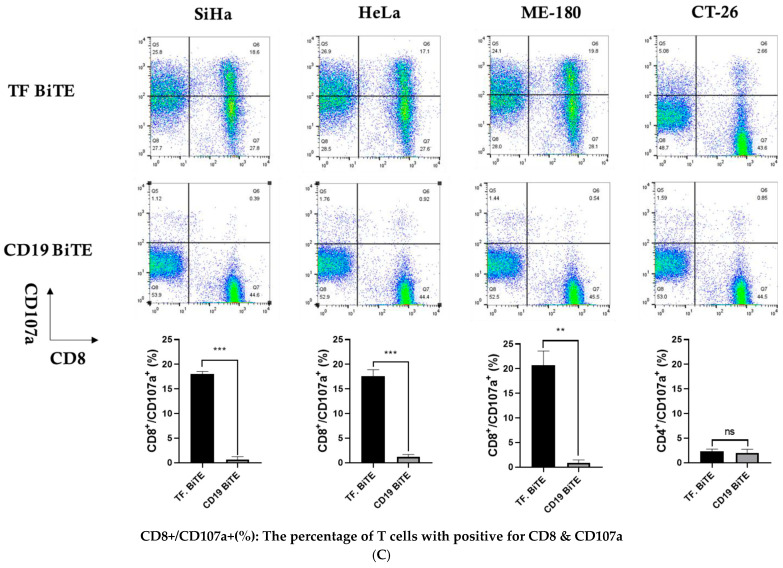
Activation and degranulation of T cells mediated by TF-BiTE. (**A**) Flow cytometric analysis of CD25 and CD69 co-expression in CD3^+^ T cells after co-culture with TF-positive tumor cells (SiHa, HeLa, ME-180, and CT-26) in the presence of TF-BiTE or control CD19-BiTE. Representative dot plots are shown for each tumor cell line, and the proportion of CD25^+^/CD69^+^ cells among total T cells is quantified in the bar graphs. TF-BiTE markedly enhanced T cell activation compared with CD19-BiTE, particularly in TF-expressing cervical cancer cells. (**B**) Measurement of degranulation in CD4^+^ T cells using CD107a surface expression. CD3^+^ T cells were incubated with the indicated tumor cell lines at an effector-to-target (E:T) ratio of 10:1 for 16 h in the presence of TF-BiTE or CD19-BiTE. Representative dot plots and bar graphs demonstrate that TF-BiTE, but not CD19-BiTE, induced significant CD107a upregulation in CD4^+^ T cells against TF-positive targets. (**C**) Degranulation of CD8^+^ T cells assessed by CD107a expression after incubation with tumor cells at an E:T ratio of 10:1 for 16 h. Representative dot plots and quantification indicate that TF-BiTE induced robust CD8^+^ T cell degranulation against TF-positive cervical cancer cell lines, whereas CT-26 cells (TF-negative control) showed no significant CD107a expression. Error bars in all graphs and figures represent the mean value with the standard error of the mean from at least three independent experiments or assays. We used ns to indicate non-significant results, whereas ** *p* ≤ 0.01 and *** *p* ≤ 0.001 indicate varying significance levels.

To compare the T-cell activation mediated by TF-BiTE with CD19-BiTE, PBMC were co-cultured with AsPC-1 cells and treated with either TF-TCB or CD19-BiTE for 16 h, followed by flow cytometry analysis. CD25 and CD69 are the most prominent markers for T-cell activation. The results showed that TF-BiTE induced T-cell activation more effectively than CD19-BiTE, as evidenced by a higher percentage of CD25^+^/CD69^+^ cells in cervical cancer cell lines. (Figure 5A).

#### 2.3.3. Degranulation Assay

The cell membrane expression of CD107a is a marker of immune cell activation and cytotoxic degranulation. Our data shows that TF-BiTE results in a similar activation of CD4^+^ and CD8^+^ T cells, as demonstrated by a percentage increase in CD107a^+^ cells in cervical cancer cell lines (Figure 5B,C). However, no activation of CD4^+^ and CD8+ T cells has been observed in CT-26 cell lines, neither by TF-BiTE nor CD19-BiTE (Figure 5B,C).

## 3. Discussion

In this study, we developed and characterized a novel bispecific T-cell engager (BiTE) targeting tissue factor (TF) and CD3 and demonstrated its potent cytotoxic activity against cervical cancer cell lines. TF was highly expressed in both squamous cell carcinoma and adenocarcinoma of the cervix, whereas it was absent or minimally detected in normal cervical tissues, supporting its potential as a selective therapeutic target. The TF-BiTE effectively redirected T cells toward cervical cancer cells, promoted proliferation, and induced cytotoxicity in vitro. These findings suggest that TF-BiTE may provide a promising immunotherapeutic strategy for cervical cancer, a disease with limited treatment options in advanced stages.

Previous studies have reported the development of BiTEs against other tumor-associated antigens, including ROR1, EpCAM, and HER2, with encouraging efficacy across solid tumors [13,14,15]. A bispecific antibody targeting TF and CD3 was previously reported by another group in 2022 [16]. Their study demonstrated antitumor activity in several TF-positive malignancies, including pancreatic and breast cancers. In contrast, our work is distinct in several important aspects. First, we focused specifically on cervical cancer, particularly the adenocarcinoma subtype, which exhibits higher TF expression than squamous carcinoma and normal cervical tissue, and where therapeutic options remain limited. Second, we conducted a comprehensive evaluation of T-cell functions, including activation (CD25/CD69), proliferation, degranulation (CD107a in CD4/CD8 subsets), and cytotoxicity, thereby providing a more detailed functional profile compared with previous studies that primarily assessed cytokine release. Third, we confirmed TF expression through archived patient tumor samples with quantitative IHC scoring, strengthening the translational rationale for cervical cancer. Taken together, these differences highlight the novelty and disease-specific relevance of our TF-BiTE construct, positioning it as a promising immunotherapeutic candidate distinct from the previously described TF/CD3 bispecific antibody.

Our study extends these findings by focusing specifically on cervical cancer, which exhibits consistently high TF expression compared with normal epithelium. We further confirmed TF expression through immunohistochemistry using a large set of archived patient tissues and demonstrated activity in both squamous and adenocarcinoma cell lines, thereby highlighting the broad applicability of TF-BiTE in different cervical cancer subtypes.

We acknowledge several limitations. Most importantly, in vivo efficacy and safety studies were not performed. Previous reports of ROR1- and TF-targeting BiTEs have demonstrated robust antitumor activity in xenograft models, underscoring the need to confirm our in vitro results in vivo. In addition, the current construct employs a rat-derived scFv, which may elicit immunogenicity in humans. Humanization and further optimization of the BiTE format will therefore be essential for clinical translation. Moreover, because the scFv sequences were generated against human TF, potential cross-reactivity with TF from preclinical species such as mouse or monkey remains uncertain. We did not directly test murine TF binding, which restricts immediate in vivo validation. Future studies will include cross-reactivity assays using mouse TF-expressing cells, xenograft models reconstituted with human PBMC, and syngeneic mouse models to evaluate full immune responses. Finally, while TF expression was validated in cervical cancer tissues, the possibility of off-tumor effects, particularly under inflammatory conditions where TF can be induced, warrants careful evaluation.

Despite these limitations, our results provide strong preclinical evidence that TF-BiTE represents an effective and selective therapeutic candidate for cervical cancer. Given the poor prognosis of recurrent or metastatic disease and the limited efficacy of current systemic therapies, TF-BiTE may offer a novel option, either as monotherapy or in combination with immune checkpoint inhibitors. This proof-of-concept study thus provides a foundation for future translational and clinical development.

Monoclonal antibodies targeting tumor-specific antigens have significantly improved outcomes in hematologic and solid tumors. Recent reviews underscore the rapid development and widespread clinical adoption of therapeutic antibodies in oncology [17]. CAR T-cell therapy has shown remarkable success in hematologic malignancies, particularly refractory B-ALL [18] but remains challenging in solid tumors due to the immunosuppressive tumor microenvironment and limited antigen availability [19]. For cervical cancer, clinical trials of TIL therapy are ongoing, but evidence for CAR-T or BiTE therapies remains scarce [20].

Targeting TF has already shown clinical relevance. Tisotumab vedotin, an antibody–drug conjugate directed against TF, demonstrated a 24% objective response rate (including 7% complete responses) in the innovaTV 204/GOG-3023/ENGOT-cx6 trial, leading to FDA approval in 2021 for recurrent or metastatic cervical cancer after chemotherapy [21]. These results highlight the therapeutic value of TF and support further exploration of alternative TF-targeted platforms such as BiTEs.

Finally, a recent study investigated a TF-BiTE across diverse solid tumor cell lines, using an IgG-[L]-scFv design rather than the tandem scFv structure employed in our construct [16]. The IgG-[L]-scFv design incorporated an IgG light chain domain, which increases molecular weight and may impair folding efficiency, stability, and tissue penetration, while potentially introducing steric hindrance. By contrast, our tandem scFv design is a more compact and manufacturable format that facilitates tissue accessibility, improves T-cell synapse formation, and enhances production yield. This structural distinction highlights the novelty of our BiTE beyond functional assays, and underscores its potential advantages for translational application.

## 4. Materials and Methods

### 4.1. Cells and Reagents

To model TF-targeted immune redirection, we utilized the human cervical carcinoma cell lines SiHa, HeLa, and ME180, and the murine colorectal cell line CT26 (used as a TF-negative control). All lines were sourced from the Korean Cell Line Bank (KCLB, Seoul, Republic of Korea), except for HEK 293T, obtained from Invitrogen (Carlsbad, CA, USA). SiHa and HeLa cells were Dulbecco’s Modified Eagle Medium (DMEM; Welgene, Gyeongsan, Republic of Korea), while ME180 was maintained in RPMI 1640 medium (Welgene, Gyeongsan, Republic of Korea). All media were supplemented with 10% heat-inactivated fetal bovine serum (FBS; Invitrogen, Carlsbad, CA, USA) and 1% penicillin-streptomycin (Invitrogen, Carlsbad, CA, USA). Cultures were maintained at 37 °C in a humidified incubator with 5% CO_2_.

For surface marker analysis, tissue factor (TF) expression was evaluated using a PE-conjugated anti-human CD142 antibody (BioLegend, San Diego, CA, USA) by flow cytometry. Cryopreserved human CD3^+^ pan-T cells (STEMCELL Technologies, Vancouver, BC, Canada) were activated using T Cell TransAct™ reagent (Miltenyi Biotec, Bergisch Gladbach, Germany) and rested prior to functional assays.

In addition, the AsPC-1 cell line (a human pancreatic adenocarcinoma line, ATCC CRL-1682) was obtained from the American Type Culture Collection (ATCC, Manassas, VA, USA) for selected experiments. Peripheral blood mononuclear cells (PBMCs) were isolated from healthy donors under institutional review board approval (IRB No. 2023-04-019, Hallym University Medical Center, Anyang, Republic of Korea), and written informed consent was obtained from all participants.

### 4.2. Patient Samples

Archived FFPE tissue blocks from 171 patients with cervical squamous cell carcinoma (103 patients) or adenocarcinoma (68 patients) were retrieved from Hallym University Medical Center pathology archive. The tissues had been collected previously with written informed consent at the time of clinical care. Subsequent use of these de-identified specimens for this research was reviewed and approved by the Institutional Review Board Hallym University Medical Center (IRB No. 2023-04-019). This study was conducted in accordance with the Declaration of Helsinki. Written informed consent was obtained from all participants.

Cases with prior chemotherapy or radiotherapy were excluded. Tissue microarrays were constructed using duplicate 2 mm tumor cores. TF immunoreactivity was visualized using DAB staining following overnight incubation with anti-CD142 antibody. Quantification was performed via H-score method by two independent pathologists, as described in previous studies [16,22]

### 4.3. Study Period

All experiments were conducted between March 2023 and February 2024 at Hallym University Medical Center and associated research laboratories.

### 4.4. Immunohistochemical Analysis of TF Expression

Tissue microarrays (TMAs) were constructed by extracting two representative 2 mm cores from each tumor block using a precision trephine instrument (Superbiochips Laboratories, Seoul, Korea). TMA sections (4 µm) were deparaffinized, rehydrated, and subjected to heat-induced epitope retrieval using citrate buffer (pH 6.0). Sections were incubated with a primary antibody against human tissue factor (CD142; 1:100 dilution, BioLegend, San Diego, CA, USA) overnight at 4 °C, followed by a biotin-free HRP detection system (EnVision™, Agilent Technologies, Santa Clara, CA, USA).

Staining was visualized using DAB chromogen and counterstained with hematoxylin. TF expression was semi-quantitatively assessed using the H-score method, which incorporates staining intensity (0–3) and the percentage of positively stained tumor cells. All slides were independently evaluated by two blind pathologists. Discordant cases were resolved by consensus.

### 4.5. Generation and Cloning of TF-BiTE

Rats were immunized against the extracellular portion of TF by Aldevron GmBH. Oligoclonal clones from the subsequent hybridomas were single cell sorted, and immunoglobulin heavy and light chain sequences were isolated by 50 reverse amplifications of cDNA ends (50 RACE) using the standard laboratory protocols. Productive sequences, as identified by the International Immunogenetics Information System V-Quest tool, 41 were cloned in frame with heavy and light chain constant regions, and antibodies were generated by transient co-transfection. Specific binding for TF was demonstrated before the conversion of the variable domains to scFvs in a heavy chain-linker-light chain format [23].

The scFvs against TF or the control CD19 (fmc63) were linked to the anti-human CD3 scFv (OKT3) using a short peptide linker. Constructs were generated by gBlocks synthesis (Integrated DNA Technologies) and assembled by overlapping extension PCR with Phusion polymerase (New England Biolabs). The resulting BiTE open reading frame (ORF) was inserted into the SFG retroviral vector upstream of a GFP reporter via NcoI/MluI sites, with the two ORFs separated by an IRES sequence. An N-terminal hexahistidine tag was included to enable detection and purification of the BiTE proteins.

### 4.6. Construction and Purification of TF-Targeted BiTE

Single-chain variable fragments (scFvs) recognizing tissue factor (TF) or the control antigen CD19 (clone FMC63) were genetically fused to an anti-human CD3 scFv (clone OKT3) through a flexible (G_4_S)_3_ linker. The TF-BiTE construct was synthesized by gBlocks (Integrated DNA Technologies) and assembled using overlapping extension PCR with Phusion polymerase (New England Biolabs). The open reading frame was inserted into an SFG retroviral expression vector upstream of an IRES-linked GFP sequence, and an N-terminal hexahistidine tag was incorporated to facilitate protein purification. Transient transfection was performed in HEK293T cells, and secreted BiTE proteins were purified from culture supernatants using Ni-NTA affinity chromatography. Protein integrity and expression were confirmed by SDS-PAGE and Western blot analysis [13].

### 4.7. In Vitro Cytotoxicity Assay

Cervical cancer target cells were labeled with CellTrace Violet (Thermo Fisher Scientific, Waltham, MA, USA) prior to co-culture to distinguish them from effector T cells. Peripheral blood T cells were used as unstimulated effectors, with anti-CD3/CD28 bead-activated T cells included in selected controls to confirm that TF-BiTE–mediated cytotoxicity was independent of pre-activation. Target cells were seeded in flat-bottom 96-well plates and allowed to adhere overnight, then co-cultured with labeled CD3^+^ T cells at effector-to-target (E: T) ratios of 1:1 to 10:1 in the presence or absence of TF-BiTE or control CD19-BiTE (1 µg/mL). After 24–36 h, cell viability was assessed using the CytoTox-Glo assay (Promega, Madison, WI, USA), and specific lysis was calculated relative to untreated controls. Each condition was tested in triplicate.

### 4.8. Flow Cytometry and Cytokine Quantification

To assess T-cell activation, co-cultured cells were stained with antibodies against CD69, CD25, and CD107a. Fluorescence data were collected on a FACSCalibur™ cytometer (BD Biosciences, San Jose, CA, USA) and analyzed with FlowJo software 10 (TreeStar, Ashland, OR, USA). Culture supernatants were collected for cytokine analysis. Levels of IFN-γ, TNF-α, and IL-2 were quantified using ELISA kits (R&D Systems, USA) by the manufacturer’s instructions.

### 4.9. Statistical Analysis

All experiments were conducted in at least three independent replicates. Statistical comparisons were performed using two-tailed Student’s *t*-tests or one-way ANOVA with Bonferroni post hoc tests, as appropriate. Data are presented as mean ± standard deviation (SD), with significance defined as *p* < 0.05.

Error bars in all graphs and figures represent the mean value with the standard error of the mean from at least three independent experiments or assays. We used NS to indicate non-significant results, whereas * *p* ≤ 0.05, ** *p* ≤ 0.01, *** *p* ≤ 0.001, and **** *p* ≤ 0.0001 indicate varying significance levels.

## Data Availability

The data that supports the findings of this study are available from the corresponding author upon reasonable request. The datasets are not publicly available due to privacy or ethical restrictions.
